# Detailed study of bifurcations in a rate model with excitatory and inhibitory neurons and adaptation

**DOI:** 10.1007/s10827-026-00944-7

**Published:** 2026-08-10

**Authors:** Anita Windisch, Péter L. Simon

**Affiliations:** 1 Department of Applied Analysis and Computational Mathematics, Institute of Mathematics, Eötvös Loránd University, Pázmány Péter sétány 1/C, 1117 Budapest, Hungary; 2 HUN-REN-ELTE Numerical Analysis and Large Networks Research Group, Budapest, Hungary

**Keywords:** rate model, adaptation, piecewise linear, bifurcation diagram, oscillation

## Abstract

The dynamical behaviour of a population-based rate model with firing adaptation is studied. An excitatory and inhibitory population of neurons is recurrently coupled and a negative feedback term is added to the excitatory population as firing adaptation. In several studies of these models, the UP-DOWN transitions are in focus, which are also exhibited by the investigated model. In this paper we provide a full characterization of equilibrium points with the precise conditions for their existence and stability. Calculations can be performed analytically and explicit formulas can be provided due to the fact that the activation function is a threshold linear function. We use bifurcation analysis to detect significant changes in the phase space. Complete list of bifurcation diagrams is provided with respect to the number of steady states. Oscillatory dynamics of neurobiological relevance are examined through local bifurcation analysis. The study demonstrates that sharp wave-ripple oscillations may emerge in certain regions of the parameter space.

## Introduction

Cortical activity displays rich state-dependent dynamics that reflect the interaction between internal network mechanisms and global brain state. One of the most notable examples is the alternation between periods of sustained firing (UP states) and quiescent periods (DOWN states). These UP-DOWN transitions have been observed during slow-wave sleep, anaesthesia, and quiet wakefulness in vivo, as well as in cortical slices in vitro (Steriade et al. 1993; Sanchez-Vives and McCormick 2000; Compte et al. 2003). They are thought to support memory consolidation by coordinating interactions between the hippocampus and neocortex, during which encoded information is repeatedly reactivated and stabilized into long-term storage. This alternating activity pattern is also believed to modulate synaptic plasticity, promoting selective changes in synaptic efficacy while preventing excessive excitation and maintaining network balance. In addition, the reduced firing rates during the DOWN state lower metabolic demand and may contribute to the energetic efficiency of cortical networks (Tukker et al. 2020; Bartram et al. 2017; Wang et al. 2021).

While these slow transitions organize large-scale cortical excitability, they also serve to gate the occurrence of faster, more localized hippocampal dynamics. Specifically, hippocampal and cortical circuits can generate transient, high-frequency events known as sharp-wave ripples (SWRs). These events occur primarily during quiescent brain states, such as non-REM sleeping and consummatory behaviours, like eating and grooming, and reflect brief episodes of highly synchronized population activity. SWRs are typically characterized by a sharp-wave component in the local field potential accompanied by fast ripple oscillations, generated by coordinated interactions between excitatory pyramidal cells and inhibitory interneurons. Computational modeling studies have shown that hippocampal sharp wave-ripples and the associated replay of neuronal sequences can emerge from structured recurrent synaptic interactions within the CA3 network. Functionally, SWRs are thought to support memory consolidation by enabling the temporally compressed replay of neuronal sequences, thus facilitating information transfer from the hippocampus to distributed cortical networks (Ecker et al. 2022; Buzsáki 2015; Gulyás and Freund 2015).

Transitions between UP and DOWN states are generally interpreted as signatures of bistable network dynamics arising from recurrent cortical circuits with strong excitation balanced by inhibition (Wilson and Cowan 1972; Amit and Brunel 1997; Latham et al. 2000). In such systems, a low-activity equilibrium (DOWN state) may coexist with a high-activity equilibrium (UP state), sustained by recurrent excitatory feedback. Slow adaptive mechanisms, such as spike-frequency adaptation or synaptic depression can promote transitions between them. In the study of Holcman and Tsodyks (2006) a mathematical model is proposed, where synaptic depression is the crucial slow variable driving transitions. When synaptic resources are depleted during the UP state, the network can no longer maintain high activity and collapses into the DOWN state. In a population based rate model proposed by Jercog et al. (2017), spike-frequency adaptation acts as a slow variable that accumulates during high-activity UP states and decays during silence. This mechanism provides activity-dependent negative feedback, effectively regulating the duration and periodicity of cortical oscillations.

Neural models that exhibit UP-DOWN transitions have been widely studied recently. In the work of Jercog et al. (2017), the authors first built a biologically based, adaptive spiking excitatory-inhibitory network capable of generating spontaneous UP-DOWN transitions. Then they introduced a 3-dimensional rate model to describe the essential dynamical structure of the spiking network, simplify the interpretation of the origin of bistability, and provide explanation of the observed state transitions. The model describes the rate dynamics of an excitatory and inhibitory population, where adaptation current is added to the excitatory network. They proved that there can exist a DOWN state equilibrium and an UP state steady state too, which are both stable in a certain domain of the parameter space resulting in bistability. The authors showed that slow adaptation acts as negative feedback term and can destabilize the UP state, thus promoting transitions back to the DOWN state. They also showed monostable regimes where only one of the UP state or DOWN state exists. When adaptation and synaptic noise are combined to induce transitions, the authors distinguished metastability, meaning that an equilibrium is stable in terms of both rates and adaptation dynamics, and quasi-stability, when the steady state is stable only for the rates, but unstable for adaptation. Synaptic noise was found essential for moving the system from the stable DOWN state into the UP state, creating the characteristic oscillatory cycle observed in the cortex. These fluctuation-driven transitions can occur for both metastable and quasi-stable states (Jercog et al. 2017).

In the work of Levenstein et al. (2019), a 2-dimensional version of the rate model built by Jercog et al. is studied by describing the neuronal population activity in terms of the mean firing rate. Activation function and also the function, that defines the level of adaptation for a given fixed rate, are taken to be sigmoidal functions. Authors showed that the model can present four distinct regimes of UP/DOWN dynamics: an oscillatory one, where the dynamics alternate between UP and DOWN states without any fluctuations; a bistable regime, where both UP and DOWN states are stable and in absence of noise, adaptation is not strong enough to induce transitions between them; and two regimes where one of the UP and DOWN states is stable, and relatively small noise can induce transitions. They also studied the model by distinguishing excitatory and inhibitory populations, but treating the adaptation variable as frozen. Power law functions were selected as activation functions and monostable and bistable regimes were determined under certain assumptions regarding parameter values.

Blum Moyse and Berry (2022) investigated how astrocytes modulate cortical UP-DOWN dynamics. They extended the rate model of Jercog et al. by introducing astrocytes as an additional dynamical component that integrates neuronal activity over slow timescales. Through numerical simulations and dynamical analysis, they showed that astrocytic feedback alters the location and extent of the bistable regime, modulates the stability of UP and DOWN fixed points, and controls the duration and frequency of state transitions.

In his dissertation, Li extended the rate model of Jercog et al. to a large-scale brain dynamics. Each area was modeled by coupled excitatory-inhibitory rate dynamics, where both populations received external inputs as well as long-range couplings. The excitatory cells further had firing adaptation, modeled by an additive negative feedback variable. Synaptic noise was introduced to model how the system transitions between rhythmic and stochastic activity. The author distinguished strong and weak adaptation regime and determined the boundary separating them. In the weak adaptation regime, both the UP and DOWN states were stable fixed points, and could switch to the opposite state in the presence of noise, while in strong adaptation regime the UP state was only transiently stable, thus dynamics returned to DOWN state by adaptation (Li 2022).

Despite these important contributions, the dynamical structure of the rate model with firing adaptation introduced by Jercog et al. has not been systematically characterized yet. The model has four steady states, whose existence and stability not only determine bistability but also contribute to the appearance of periodic solutions. Moreover, the system contains eleven parameters that significantly affect its dynamical behaviour, making the global description challenging. For this reason, our primary goal is to understand the local dynamics of the system by determining equilibria with precise conditions for their existence and analyzing their stability. Then based on this, our aim is to understand the occurrence of periodic behaviour.

The paper is structured as follows. In Section 2, we introduce the rate model we study and also the methods used for analyzes. In the first part of Section 3, we calculate the four equilibria of the system with the precise conditions for their existence, and study their stability. Then, in Subsection 3.2, fold bifurcation curves are determined and the complete list of bifurcation diagrams are shown for any parameter setting with the number of equilibria in each domain. In Subsection 3.3, we study the occurrence of limit cycles via focus-center-limit cycle bifurcation and investigate the dynamics of the system in detail for a given parameter set. Finally, in Section 4, we discuss our results and outline possible directions for future work.

## Model equations and methods

The section begins with a description of the population-based rate model developed by Jercog et al. (2017), followed by the modifications implemented for the present study. We subsequently introduce the mathematical methods employed to analyze the model.

Mathematical models of populations of neurons start from the pioneering model developed by Wilson and Cowan (1972). Considering a population of excitatory and inhibitory neurons the model can be formulated as a system of two ordinary differential equations. This can be extended by adaptation of the neurons. Here we present the three-variable model introduced by Jercog et al. (2017), and investigate its dynamics. This model describes the firing rate of an excitatory ($r_E$) and inhibitory ($r_I$) population which are recurrently connected, and a negative feedback term (*a*) is added to the excitatory population, representing the firing adaptation. In the original three-dimensional model, the external inputs contain the fluctuating parts $\sigma \xi _E(t)$ and $\sigma \xi _I(t),$ modeled as independent Ornstein-Uhlenbeck processes, which we remove from the model by setting *σ =0* in order to investigate the dynamical behaviour in absence of noise. These noise-free equations are subject of bifurcation analysis and determine the bifurcation diagram. The bifurcation curves divide the parameter region into several subregions where the system shows different dynamical behaviour. In some parameter regions the system shows multistability, i.e. different behaviours can be observed depending on the initial condition. When noise is added to the system, it may cause a trajectory to move from the basin of attraction of one steady state or periodic orbit to another one. This could lead a trajectory to visit different attractors

1 $$\begin{aligned} \tau _E\dot{r_E}= & -r_E+\varphi _E\left( w_{EE}r_E-w_{EI}r_I-a \right), \nonumber \\ \tau _I\dot{r_I}= & -r_I+\varphi _I\left( w_{IE}r_E-w_{II}r_I \right), \\ \tau _a\dot{a}= & -a+\beta r_E\nonumber \text {.} \end{aligned}$$

The transfer functions $\varphi _E$, $\varphi _I$ are modeled by the threshold-linear functions

$$\begin{aligned} \varphi _E(x)=g_E\left[ x-\theta _E\right] _{+} \quad \text {and}\quad \varphi _I(x)=g_I\left[ x-\theta _I\right] _{+}, \end{aligned}$$

where $\left[ x-\theta _X\right] _{+}=x-\theta _X$ if $x-\theta _X>0$, and $x-\theta _X=0$ otherwise, for $X\in \left\{ E,I\right\}$. The effective thresholds $\theta _E$ and $\theta _I$ denote the difference between the activation threshold minus the mean external current, while $g_E$, $g_I$ represent gains, which refer to the sensitivity of the excitatory and inhibitory populations to their input. The original model describes the synaptic couplings by $J_{XY}>0$ for $X,Y \in \{E,I\}$ denoting the strength of connections from *Y* to *X*. Here we introduce the general parameter of connection strength *c* with $w_{XY}= c J_{XY}$ for $X,Y\in \{ E,I \}$, and choose it as control parameter, together with *β*, which represents the strength of adaptation. The two rates and also the adaptation have different time constants $\tau _E, \tau _I, \tau _a$. 

In this work, our aim is to provide full characterization of the parameter space according to the dynamical behaviour of the model, which we do in two main steps. First, the local behaviour is studied by determining steady states and the domains in the parameter space where they exist, then their stability is analyzed. Based on this, we investigate the occurrence of periodic orbits.

Since the model is a system of piecewise linear differential equations, many properties can be calculated analytically. Explicit formulas for equilibria in terms of all parameters can be provided, and also the conditions for their stability. Bifurcation analysis is used to detect significant changes in the parameter space. Local bifurcations, such as fold bifurcation, and birth of a periodic orbit related to pure imaginary eigenvalues of the Jacobian matrix, are also determined analytically.

## Results

### Steady states and their stability

In the literature of adaptation-driven rate models, generally UP and DOWN steady states are in focus. Here we show a full characterization of steady states. Moreover, we give the precise conditions for their existence and stability in the high dimensional parameter space.

#### Existence of steady states

The steady states of system (1) are given by the system

2 $$\begin{aligned} -r_E+g_E\left[ cJ_{EE}r_E-cJ_{EI}r_I-a-\theta _E\right] _+= & 0, \nonumber \\ -r_I+g_I\left[ cJ_{IE}r_E-cJ_{II}r_I-\theta _I\right] _+= & 0,\\ -a+\beta r_E= & 0,\nonumber \end{aligned}$$

where the definitions of all parameters are given below equations (1). Four cases will be distinguished according to the signs of the expressions in $[ \cdot ]_+$. Substituting $a=\beta r_E$ into the first equation, we study the four cases in a straightforward way below.

**Case 1:** $$(c_{JEE}-\beta )r_E-c_{JEI}r_I-\theta _E\le 0$$ and $$c_{JIE}r_E-c_{JII}r_I-\theta _I\le 0$$

The steady state equations yield $r_E=0$, $r_I=0$ and *a=0*. We need to check whether the two conditions hold with these values of $r_E$ and $r_I$. Substituting $r_E=0$ and $r_I=0$ into the conditions yields $\theta _E\ge 0$ and $\theta _I\ge 0$. Hence we have the following statement.

##### Proposition 1

The steady state $r_E=0$, $r_I=0$, *a=0* exists if and only if $\theta _E\ge 0$ and $\theta _I\ge 0$.

It is useful to introduce the notations $d_E:=g_E c J_{EE}-1$ and $d_I:=g_I c J_{II}+1$ in order to calculate the steady states in the following cases.

**Case 2:** $$\left( cJ_{EE}-\beta \right) r_E-cJ_{EI}r_I-\theta _E \le 0$$ and $$c J_{IE}r_E-c J_{II}r_I-\theta _I \ge 0$$

The steady state equations yield $r_E=0$, $r_I=-\frac{g_I\theta _I}{d_I}$ and *a=0*. We need to check whether the two conditions hold with these values of $r_E$ and $r_I$. Substituting $r_E=0$ and $r_I=-\frac{g_I\theta _I}{d_I}$ into the conditions yields $\theta _I g_I c J_{EI} \le d_I \theta _E$ and $\theta _I\le 0$. We may conclude the following.

##### Proposition 2

The steady state $r_E=0$, $r_I=-\dfrac{g_I\theta _I}{d_I}$, *a=0* exists if and only if $\theta _I g_I c J_{EI} \le d_I \theta _E$ and $\theta _I\le 0$.

**Case 3**: $$\left( cJ_{EE}-\beta \right) r_E-cJ_{EI}r_I-\theta _E \ge 0$$ and $$c J_{IE}r_E-c J_{II}r_I-\theta _I \le 0$$

The steady state equations yield

$$\begin{aligned} r_E=\dfrac{g_E\theta _E}{d_E-g_E\beta }, \quad r_I=0, \quad a=\beta \dfrac{g_E\theta _E}{d_E-g_E\beta }. \end{aligned}$$

We need to check whether the two conditions hold with these values of $r_E$ and $r_I$. Substituting the values into the conditions yields

3 $$\begin{aligned} \left( d_E-g_E\beta \right) \theta _E\ge 0 \quad \text{ and } \quad \theta _I\ge \dfrac{g_E c J_{IE}}{d_E-g_E\beta }\theta _E. \end{aligned}$$

As a consequence, we have the following.

##### Proposition 3

The steady state $r_E=\dfrac{g_E\theta _E}{d_E-g_E\beta }$, $r_I=0$, $a=\beta \dfrac{g_E\theta _E}{d_E-g_E\beta }$ exists if and only if the conditions in equation (3) hold.

**Case 4**: $$\left( cJ_{EE}-\beta \right) r_E-cJ_{EI}r_I-\theta _E \ge 0$$ and $$c J_{IE}r_E-c J_{II}r_I-\theta _I \ge 0$$

The steady state equations yield

$$\begin{aligned} r_E=g_E\dfrac{{A}}{{N}}, \quad r_I=g_I\dfrac{{B}}{{N}}, \quad a=\beta g_E\dfrac{{A}}{{N}}, \end{aligned}$$

where

4 $$\begin{aligned} A:= & g_I c J_{EI}\theta _I-\theta _E d_I\text {,}\nonumber \\ B:= & \theta _I d_E-g_E c J_{IE}\theta _E-g_E\theta _I\beta \text {,}\nonumber \\ {N}:= & g_Eg_I c^2 J_{EI}J_{IE}-d_Ed_I+g_Ed_I\beta \nonumber \text {.} \end{aligned}$$

We need to check whether the two conditions hold with these values of $r_E$ and $r_I$. Substituting the values into the conditions yields ${N}>0$, ${A}\ge 0$, ${B}\ge 0$; or ${N}<0$, ${A}\le 0$, ${B}\le 0$. Thus, we obtain the following result.

##### Proposition 4

The steady state $r_E=g_E\dfrac{{A}}{{N}}$, $r_I=g_I\dfrac{{B}}{{N}}$, $a=\beta g_E\dfrac{{A}}{{N}}$ exists if and only if the conditions ${N}>0$, ${A}\ge 0$, ${B}\ge 0$; or ${N}<0$, ${A}\le 0$, ${B}\le 0$ hold. 

##### Remark 1

Jercog et al. (2017) demonstrated the existence of a bistable regime consisting of stable UP and DOWN states separated by an unstable equilibrium. However, the parameter dependence of the equilibria was not investigated systematically. In the present work, explicit existence conditions are derived for all four equilibria, providing a complete characterization of the parameter regions in which each equilibrium exists.

The obtained existence regions show how adaptation affects the possible activity states of the network. In particular, Cases 2 and 3 show that adaptation can create equilibrium states in which only one of the two populations remains active, thereby extending the range of possible network behaviors beyond the classical UP and DOWN states.

#### Stability of the steady states

Now, we determine the stability of the steady states in the cases above by using linearization. In each case, we calculate the Jacobian at the given steady state and then we determine the conditions for all eigenvalues having negative real part. These conditions are formulated in terms of the parameters, leading to bifurcation curves along which the stability of a given equilibrium changes. System (1) is piecewise linear, hence its Jacobian is identical to the right hand side of the system (after division by the time constants). However, it has to be kept in mind that the right hand side depends on the signs of the quantities in the arguments of the *φ* functions. Thus, we calculate the Jacobian separately for each case, where the signs of these quantities are given.

**Case 1**: The Jacobian matrix at the steady state $r_E=0$, $r_I=0$, *a=0* is

$$J = \begin{bmatrix} -\frac{1}{\tau _E}& 0& 0\\ 0& -\frac{1}{\tau _I}& 0\\ \frac{\beta }{\tau _a}& 0& -\frac{1}{\tau _a} \end{bmatrix}$$

Its eigenvalues are $\lambda _{1}=-\frac{1}{\tau _E}$, $\lambda _{2}=-\frac{1}{\tau _I}$ and $\lambda _{3}=-\frac{1}{\tau _a}$, hence this equilibrium is stable (once it exists) since all eigenvalues are negative. 

**Case 2**: The Jacobian matrix at the steady state $r_E=0$, $r_I=-\dfrac{g_I\theta _I}{d_I}$, *a=0* is

$$J = \begin{bmatrix} -\frac{1}{\tau _E}& 0& 0\\ \frac{g_I c J_{IE}}{\tau _I}& -\frac{d_I}{\tau _I}& 0\\ \frac{\beta }{\tau _a}& 0& -\frac{1}{\tau _a} \end{bmatrix}$$

Its eigenvalues are $\lambda _{1}=-\frac{1}{\tau _E},$ $\lambda _{2}=-\frac{d_I}{\tau _I}$ and $\lambda _{3}=-\frac{1}{\tau _a},$ hence this equilibrium is stable (once it exists) since all eigenvalues are negative. 

**Case 3**: The Jacobian matrix at the steady state

$$\begin{aligned} r_E=\dfrac{g_E\theta _E}{d_E-g_E\beta }, \quad r_I=0, \quad a=\beta \dfrac{g_E\theta _E}{d_E-g_E\beta } \end{aligned}$$

is

$$J = \begin{bmatrix} \frac{d_E}{\tau _E}& -\frac{g_E c J_{EI}}{\tau _E}& -\frac{g_E}{\tau _E}\\ 0& -\frac{1}{\tau _I}& 0\\ \frac{\beta }{\tau _a}& 0& -\frac{1}{\tau _a} \end{bmatrix}$$

Its eigenvalues are $\lambda _{1}=-\dfrac{1}{\tau _I}<0$ and $\lambda _{2},$ $\lambda _{3},$ that are solutions of equation

$$\begin{aligned} \lambda ^2 +\left( \dfrac{1}{\tau _a}-\dfrac{d_E}{\tau _E}\right) \lambda -\dfrac{g_E\beta -d_E}{\tau _E\tau _a}=0 . \end{aligned}$$

The stability of this equilibrium may change when the real part of the roots becomes zero that happens at

$$\begin{aligned} \left( \dfrac{1}{\tau _a}-\dfrac{d_E}{\tau _E}\right) =0, \end{aligned}$$

that is when

5 $$\begin{aligned} g_E c J_{EE}=\dfrac{\tau _E}{\tau _a}+1 . \end{aligned}$$

**Case 4**: The Jacobian matrix at the steady state

$$\begin{aligned} r_E=g_E\dfrac{A}{N}, \quad r_I=g_I\dfrac{B}{N}, \quad a=\beta g_E\dfrac{A}{N} \end{aligned}$$

is

$$J = \begin{bmatrix} \frac{d_E}{\tau _E}& -\frac{g_E c J_{EI}}{\tau _E}& -\frac{g_E}{\tau _E}\\ \frac{g_I c J_{IE}}{\tau _I}& -\frac{d_I}{\tau _I}& 0\\ \frac{\beta }{\tau _a}& 0& -\frac{1}{\tau _a} \end{bmatrix}$$

Its eigenvalues are solutions of the characteristic equation $\lambda ^3-b_2 \lambda ^2 + b_1 \lambda -b_0 =0,$ where $b_0=\det J,$ $b_2=\text{ tr } J$ and $b_1=J_{11}+J_{22}+J_{33}$, where $J_{ii}$ denote the sub-determinants belonging the diagonal entries of *J*. The stability of this equilibrium may change when the real part of the roots becomes zero that happens at

$$\begin{aligned} b_0-b_1b_2=0 \quad \text {and } \quad b_1>0\text {,} \end{aligned}$$

that is when

6 $$\beta = \dfrac{\begin{array}{c} \tau _a^2 (d_E \tau _I- d_I \tau _E)(g_Eg_I c^2 J_{EI}J_{IE} - d_Ed_I) \\- \tau _I^2 d_E (d_E\tau _a-\tau _E) - \tau _E^2 d_I (d_I\tau _a+\tau _I)\end{array}}{\tau _I^2 g_E (\tau _E - d_E\tau _a)}$$

and

7 $$\begin{aligned} \beta> \dfrac{d_Ed_I\tau _a+d_E\tau _I-d_I\tau _E-g_Eg_I c^2 J_{EI}J_{IE}\tau _a}{g_E\tau _I} .\end{aligned}$$

##### Remark 2

Jercog et al. (2017) showed that the system can exhibit bistability, consisting of stable UP and DOWN states (corresponding to Cases 4 and 1, respectively, in our classification) separated by an unstable equilibrium. They identified the UP state as an inhibition-stabilized state and showed that adaptation shifts the excitatory nullcline, potentially eliminating either the UP or the DOWN state and thereby leading to monostability. They also distinguished between metastable and quasi-stable states, emphasizing that transitions between UP and DOWN states in the metastable regime are driven by stochastic fluctuations rather than adaptation alone. The present work provides a complete stability analysis of all four equilibria of the system. The stability properties are investigated with respect to all model parameters, and explicit conditions are derived for the changes in stability as functions of the parameters. While Jercog et al. (2017) focused primarily on the dynamics of bistability and state transitions, here the emphasis is placed on the parameter dependence of the equilibria and their stability.

Changes in the stability of the equilibria are closely related to qualitative changes in the network dynamics. In particular, stability loss may lead to the emergence of oscillatory activity, when a periodic orbit becomes the attracting solution. Experimental evidence also suggests that slow adaptation-related currents can contribute to the emergence of oscillatory activity; for example, Bevan and Wilson (1999) demonstrated spontaneous rhythmic firing in subthalamic neurons generated by the interaction of intrinsic membrane currents. Therefore, the stability analysis helps to identify parameter regions associated with stationary activity, bistability, or oscillatory behaviour.

### Local bifurcation diagrams

Our goal here is to determine the local bifurcation diagrams, that is to divide the parameter space according to the number of steady states. As it was mentioned in Section 2, our control parameters are the general strength of connections, characterized by the parameter *c* and the effect of adaptivity, described by the parameter *β*. We will draw the bifurcation diagrams in the *(c, β )* parameter plane. It is important to note that both control parameters are positive, hence the bifurcation curves will be drawn in the positive quadrant of the plane. Besides these two parameters, we have 11 other parameters, namely, four connection parameters, $J_{EE}, J_{EI}, J_{IE}, J_{II}$, four parameters of the non-linearity, $g_E, g_I, \theta _E, \theta _I$, and three time constants, $\tau _E, \tau _I, \tau _a$. It is important to note that the parameters listed above are positive, except for $\theta _E, \theta _I$, which can take both positive and negative values. The bifurcation diagram in the *(c, β )* parameter plane of control parameters may change as the values of these other 11 parameters are varied. Some typical values of these parameters are available in the literature, however, it turned out that the bifurcation diagram can significantly change, hence we will give a full list of bifurcation diagrams that can occur for different values of these parameters. The parameter values are based on typical values reported in the literature, with some modifications. These values are used only for the numerical computation of periodic orbits and are listed in equation (15).

The conditions for the existence of different steady states have already been determined and presented in the propositions in the previous subsection. Given the values of all parameters, the propositions determine if the actual steady state exists or not, that is for a given parameter set we can determine the exact number of steady states. The problem here appears in the opposite way. Divide the plane of control parameters according to the number of equilibria, i.e. determine the fold bifurcation curves. Bifurcation curves are discussed in some studies, but different parameter values are fixed at some neurobiologically validated values. Numerical experiments show that varying the parameter values even slightly, new behaviours may occur. In order to reveal the possible dynamics, we let all parameters vary and determine all possible shapes of the bifurcation diagrams.

#### Local bifurcation curves

Let us consider first the steady state $r_E=0$, $r_I=0$, *a=0* considered in Case 1. The existence of this equilibrium does not depend on the control parameters *c* and *β*, hence there will be no bifurcation curve corresponding to this steady state, however, it will contribute to the total number of equilibria if and only if $\theta _E\ge 0$ and $\theta _I\ge 0$, see Proposition 1.

The steady state $r_E=0$, $r_I=-\dfrac{g_I\theta _I}{d_I}$, *a=0*, considered in Case 2 will yield a bifurcation curve. Conditions for the existence of this equilibrium are $\theta _I\le 0$ and $\theta _I g_I c J_{EI} \le d_I \theta _E$, see Proposition 2. Therefore its existence does not depend on *β*, hence the bifurcation curve will be the vertical line $\theta _I g_I c J_{EI} = (g_I c J_{II}+1)\theta _E$. This leads to the following.

##### Proposition 5

The bifurcation curve determined by the equilibrium corresponding to Case 2 can be expressed as

8 $$\begin{aligned} g_I c (\theta _I J_{EI}-\theta _E J_{II}) = \theta _E . \end{aligned}$$

If $\theta _I\le 0$, depending on the sign of $\theta _E$ and $\theta _I J_{EI}-\theta _E J_{II}$, this steady state contributes to the total number of equilibria when *(c, β )* is in the left or right hand side of this vertical line.

The steady state $r_E=\dfrac{g_E\theta _E}{d_E-g_E\beta }$, $r_I=0$, $a=\beta \dfrac{g_E\theta _E}{d_E-g_E\beta }$, considered in Case 3 yields a more interesting bifurcation curve, still being a line. The condition for the existence of this equilibrium is the system of inequalities in equation (3), see Proposition 3. We need to express these inequalities in terms of the control parameters *c* and *β.* A direct consequence of the inequalities is $\theta _I>0$ and that $\theta _E$ and $d_E-g_E\beta = g_E c J_{EE}-1-g_E\beta$ have the same sign. Thus we can distinguish two cases. If $\theta _E>0$, then $d_E-g_E\beta>0$, hence the second inequality can be multiplied by this term leading to

$$c \frac{J_{EE}\theta _I- J_{IE}\theta _E}{\theta _I} - \frac{1}{g_E} \ge \beta .$$

If $\theta _E<0$, then $d_E-g_E\beta <0$, hence the second inequality leads to

$$c \frac{J_{EE}\theta _I- J_{IE}\theta _E}{\theta _I} - \frac{1}{g_E} \le \beta .$$

Observe that the first inequality is equivalent to $\theta _I (d_E-g_E\beta ) \ge g_E c J_{IE} \theta _E$. Hence assuming that this inequality holds together with $\theta _E>0$ and $\theta _I>0$ we have that $d_E-g_E\beta>0$, i.e. the first inequality of (3) holds as well. Summarizing, the equilibrium corresponding to Case 3 exists if and only if one of the following two condition holds.

9 $$\begin{aligned} \theta _E>0, \quad \theta _I>0, \quad c \frac{J_{EE}\theta _I- J_{IE}\theta _E}{\theta _I} - \frac{1}{g_E} \ge \beta , \end{aligned}$$

or

10 $$\begin{aligned} \theta _E<0, \quad \theta _I>0, \quad c \frac{J_{EE}\theta _I- J_{IE}\theta _E}{\theta _I} - \frac{1}{g_E} \le \beta . \end{aligned}$$

This leads to the following statement. 

##### Proposition 6

The bifurcation curve corresponding to the equilibrium in Case 3 is the line

11 $$\begin{aligned} c g_E(J_{EE}\theta _I- J_{IE}\theta _E) - \theta _I = \beta \theta _I g_E . \end{aligned}$$

Depending on the sign of $\theta _E$ and that of the coefficient of *c*, this steady state contributes to the total number of equilibria when *(c, β )* is below or above this line.

The steady state corresponding to Case 4 determines three bifurcation curves, *A=0*, *B=0* and *N=0*. Simple calculation shows that *A=0* is equivalent to equation (7) and *B=0* is equivalent to equation (10). Therefore, we arrive at the following statement.

##### Proposition 7

The equilibrium corresponding to Case 4 introduces only one new bifurcation curve *N=0* that can be expressed in terms of the control parameters as

12 $$\begin{aligned} c g_E J_{EE} -1 - \frac{c^2 g_E g_I J_{EI}J_{IE}}{c g_I J_{II}+1} = \beta g_E . \end{aligned}$$

It is interesting to note that the three bifurcation curves have a common point. The results above can be summarized as follows.

##### Theorem 8

The number of steady states of system (2) is determined by three bifurcation curves (7), (10) and (11) in the *(c,β )* plane of the control parameters.

##### Remark 3

Equation (10) was also calculated by Jercog et al. (2017), however we determine here all bifurcation curves that yield changes in the number of equilibrium points or in the Cases they correspond to.

#### Complete list of bifurcation diagrams

The mutual position of the three bifurcation curves is determined by the parameters, of which only $\theta _E$ and $\theta _I$ can be negative. Here we list all possible bifurcation diagrams that may occur for different parameter settings. The conditions of Theorem 8 show that the values of $\theta _E$ and $\theta _I$ play an important role in the position of the three bifurcation curves (7), (10) and (11), which together form the bifurcation diagrams. We will show that the parameter plane $(\theta _E,\theta _I)$ can be divided into 8 domains separating different cases for which we list all mutual positions of the three bifurcation curves in the *(c,β )* plane of the control parameters. This means that for each domain, a set of bifurcation diagrams is determined independently of the values of the other parameters, i.e. varying the values of the other parameters will not result in a new bifurcation diagram.

**Fig. 1:**
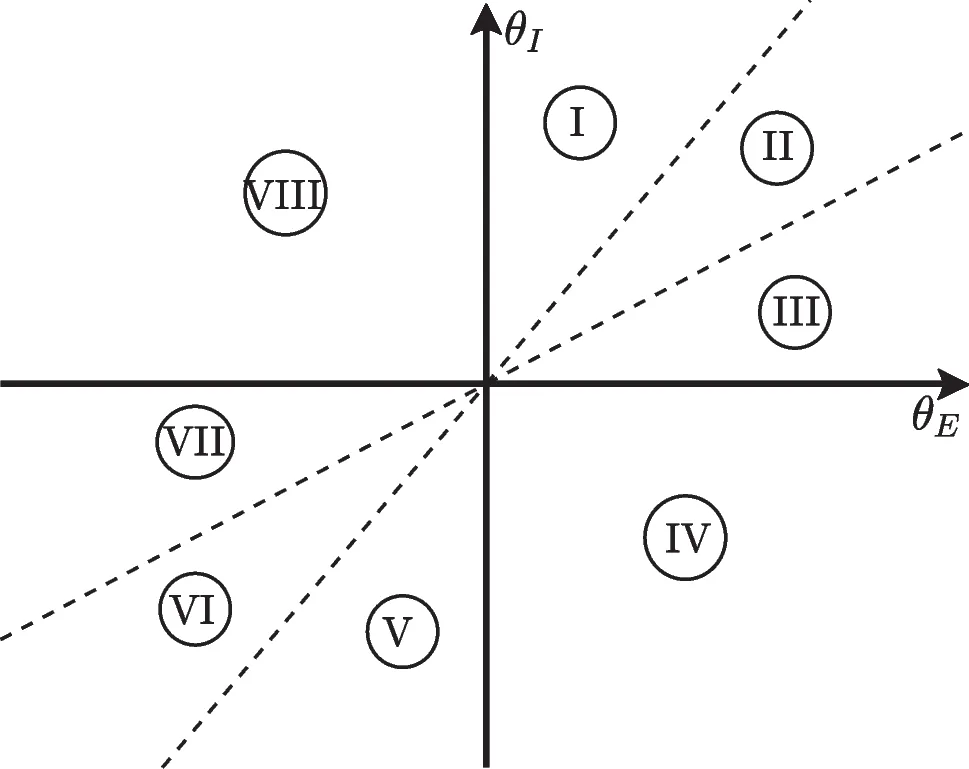
The $(\theta _{E},\theta _{I})$ plane divided into 8 domains according to the bifurcation diagrams. The dashed lines are given by $\theta _I=\alpha _1 \theta _E$ and $\theta _I=\alpha _2 \theta _E$. The domains are distinguished based on the signs of $\theta _E$ and $\theta _I$, together with the following conditions: I: $b_E,b_I>0$; II: $b_Eb_I<0$; III: $b_E,b_I<0$; IV: $\theta _E>0$, $\theta _I<0$; V: $b_E,b_I<0$; VI: $b_Eb_I<0$; VII: $b_E,b_I>0$ and VIII: $\theta _E<0$, $\theta _I>0$. Bifurcation diagrams connecting to each domain are listed in Theorem 9

**Fig. 2:**
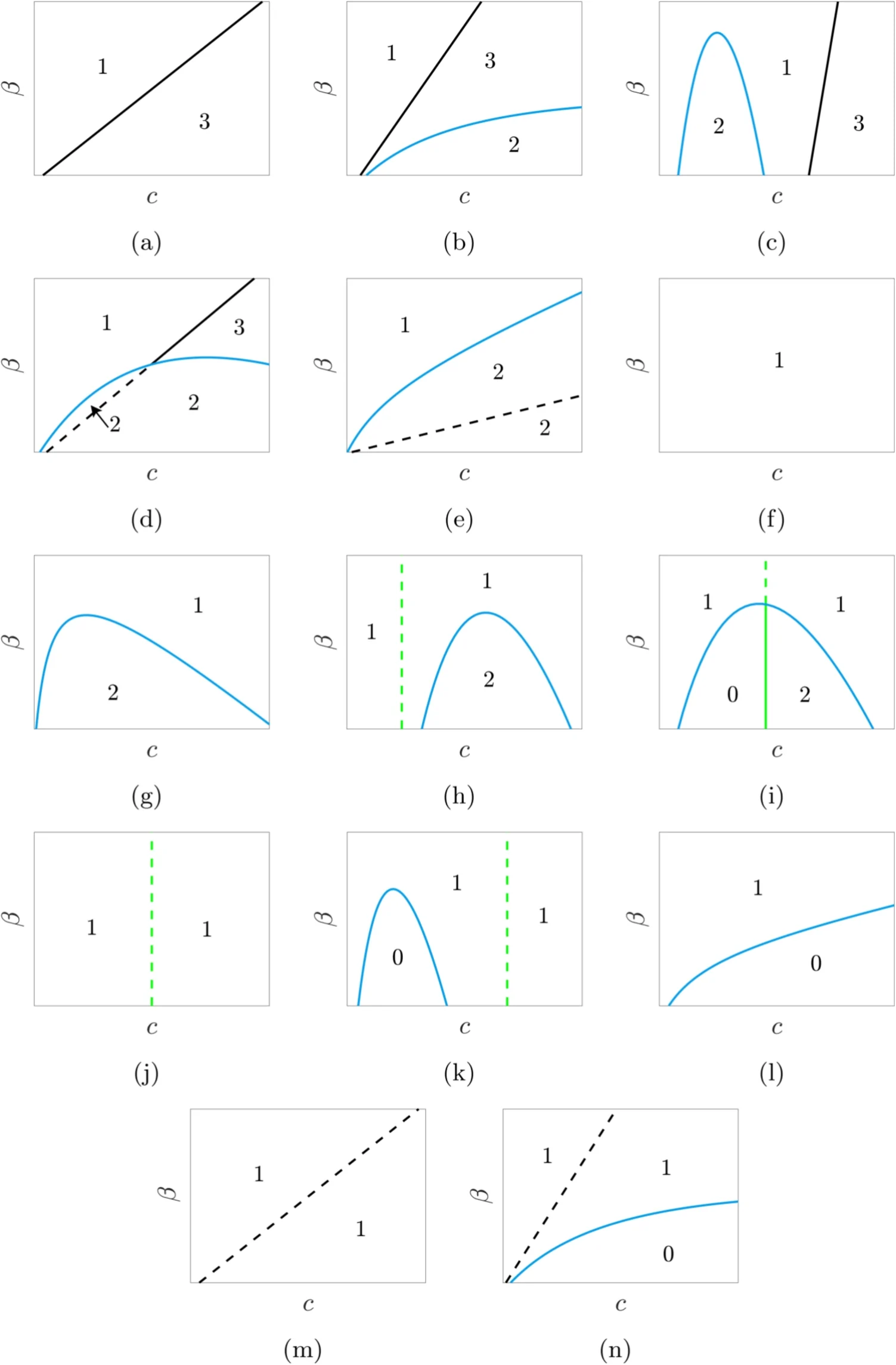
List of all possible bifurcation diagrams. Line (7) is plotted by green, line (10) by black and curve (11) by blue. Solid curves show fold bifurcations, dashed lines indicate changes in the cases that the steady states are connected to. The number of equilibria is shown for each domain. The scales of the bifurcation diagrams are not shown because they depend strongly on the values of the other parameters

Now we introduce notations

13 $$\begin{aligned} b_E&:=J_{EE}\theta _I-J_{IE}\theta _E,\\ b_I&:=J_{EI}\theta _I-J_{II}\theta _E \nonumber \end{aligned}$$

in order to define the domains and detect the bifurcation diagrams. It can be seen that $b_E>0$ if and only if $\theta _I>\dfrac{J_{IE}}{J_{EE}}\theta _E$, which can be written as $\theta _I>\alpha _1\theta _E$ if $\alpha _1:=\dfrac{J_{IE}}{J_{EE}}$ is introduced. Similarly, $b_I>0$ if and only if $\theta _I>\dfrac{J_{II}}{J_{EI}}\theta _E$, which can be written as $\theta _I>\alpha _2\theta _E$, where $\alpha _2:=\dfrac{J_{II}}{J_{EI}}$. Here, $\alpha _1$ and $\alpha _2$ are the slopes of the lines determining the change in sign of $b_E$ and $b_I$. Two cases can be distinguished according to the mutual position of these separating lines. First, $\alpha _1>\alpha _2$ if and only if $J_{EI}J_{IE}<J_{EE}J_{II}$, which is equivalent to $\det \tilde{J}>0$, where $$\tilde{J}=\begin{bmatrix} J_{EE} & J_{EI}\\ J_{IE} & J_{II} \end{bmatrix}$$ is the matrix of synaptic couplings. The other case is when $\alpha _1<\alpha _2$, which is equivalent to $J_{EI}J_{IE}>J_{EE}J_{II}$, that is $\det \tilde{J}<0$. Now merging the two cases when $b_E$ and $b_I$ have opposite signs, the $\left( \theta _E,\theta _I\right)$ plane can be divided into 8 domains by the lines denoting the change in sign of $\theta _E, \theta _I, b_E, b_I$, as it is shown in Fig. 1. The second and fourth quadrant of the parameter plane form separate domains, while three additional subdomains $b_E>0, b_I>0$; $b_Eb_I<0$ and $b_E<0, b_I<0$ are distinguished in the first and third quadrants, where $b_E$ and $b_I$ are defined in equation (12). Results with the list of all possible bifurcation diagrams for each domain are described in Theorem 9, then illustrated in Fig. 1, denoted by letters of subfigures of Fig. 2. We present in detail the structure of the bifurcation diagrams in Fig. 2. The solid lines and curves are the fold bifurcation curves which divide the *(c,β )* parameter plane into several domains where the number of steady states differs. This means that choosing parameter values from the same domain leads to the same number of equilibria, while crossing any of these bifurcation curves results in a change in the number of steady states. It may happen that choosing different parameter values from the same domain gives the same number of equilibria, but these steady states are connected to different cases listed in Section 3.1. This change that occurs in the Cases is plotted by dashed lines in the subfigures of Fig. 2. The number of steady states is also shown for each domain in Fig. 2.

##### Theorem 9

Let the subdomains I, II, ..., VIII be determined by the lines $\theta _I=\alpha _1\theta _E$ and $\theta _I=\alpha _2\theta _E$ as shown in Fig. 1.

(i) If $\left( \theta _E,\theta _I\right)$ is in domain I, then there can be 4 different bifurcation diagrams. These are shown in Fig. 2a, 2b, 2c and 2d.
(ii) If $\left( \theta _E,\theta _I\right)$ is in domain II, then there can be 3 different bifurcation diagrams. In the case $\det \tilde{J}>0$ this is Fig. 2e, while in the case of $\det \tilde{J}<0$ these are 2f and 2g.
(iii) If $\left( \theta _E,\theta _I\right)$ is in domain III, then there can be 2 different bifurcation diagrams. These are shown in Fig. 2f and 2g.
(iv) If $\left( \theta _E,\theta _I\right)$ is in domain IV, then there can be 2 different bifurcation diagrams. These are shown in Fig. 2f and 2g.
(v) If $\left( \theta _E,\theta _I\right)$ is in domain V, then there can be 4 different bifurcation diagrams. These are shown in Fig. 2h, 2i, 2j and 2k.
(vi) If $\left( \theta _E,\theta _I\right)$ is in domain VI, then there can be 3 different bifurcation diagrams. In the case $\det \tilde{J}>0$ these are shown in Fig. and 2j and 2k, while in the case of $\det \tilde{J}<0$ it is 2l.
(vii) If $\left( \theta _E,\theta _I\right)$ is in domain VII, then there can be 2 different bifurcation diagrams. These are shown in Fig. 2f and 2l.
(viii) If $\left( \theta _E,\theta _I\right)$ is in domain VIII, then there can be 2 different bifurcation diagrams. These are shown in Fig. 2m and 2n.

The detailed proof of Theorem 9 is presented in the Appendix, in which we use the conditions for the sign of the parameters, and also that the curve given in equation (11) is concave for all *c≥ 0*, and the three bifurcation curves have a common point.

### Bifurcations leading to periodic orbits

Having investigated the local behaviour of the system in Sections 3.1-3.2, our goal now is to extend the study to oscillations. Stability of steady states was determined by using linearization and it turned out that equilibria corresponding to Case 3 and 4 can lose their stability. Those parameter values at which the stability change can happen were given explicitly in equations (4) and (5). These formulas not only determine the change in stability, but also provide the necessary conditions for focus-center-limit cycle bifurcation. In this section we will review how this bifurcation can occur in a piecewise linear system, then give the list of bifurcation diagrams in the parameter plane $\left( c,\beta \right)$ while a connection parameter $J_{EE}$ is also chosen as bifurcation parameter. The dynamical behaviour of the system will be examined in detail for a chosen parameter set with the addition of bifurcation diagrams and phase portraits.

#### The focus-center-limit cycle bifurcation

**Fig. 3:**
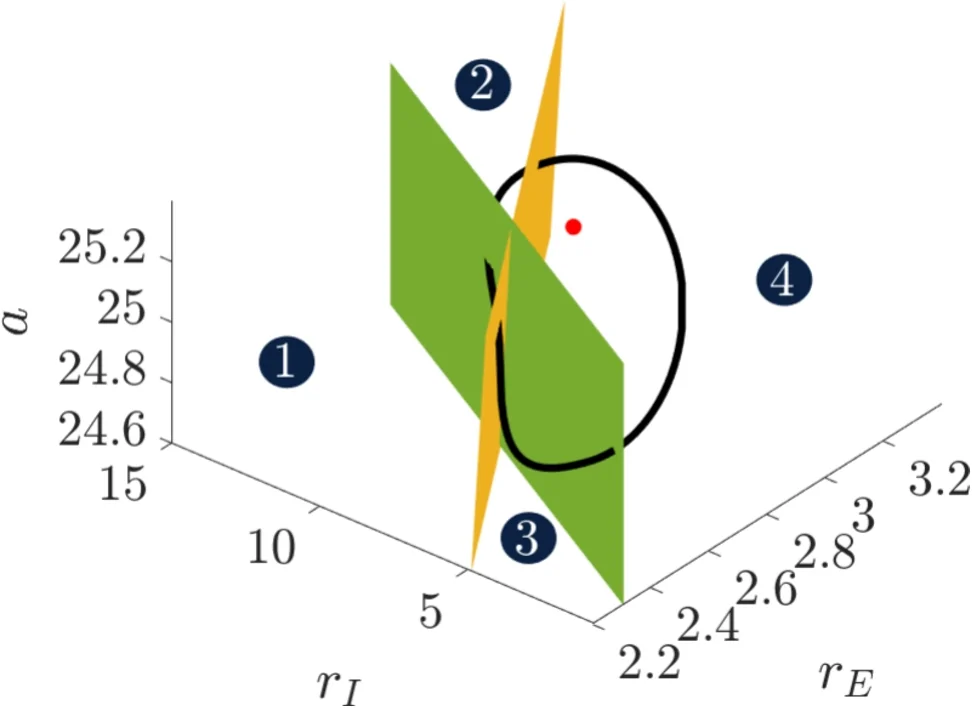
Stable periodic solution passes through the 4 domains created by the separation planes (13)-(14) of the piecewise linear system (1) with the unstable steady state of Case 4

In a piecewise linear differential system, trajectories that pass through at least 2 domains created by the separation planes in the phase space, can form periodic orbits if they intersect themselves. As an example, Figure 3 shows a solution of the studied system, that forms a periodic orbit passing all 4 domains of the phase space created by the separation planes

14 $$\begin{aligned} cJ_{EE}r_E-cJ_{EI}r_I-a= & 0 \quad \text {(orange),} \\ cJ_{IE}r_E-cJ_{II}r_I= & 0 \quad \text {(green).} \end{aligned}$$

Next, we consider the bifurcation induced by two eigenvalues of the Jacobian matrix crossing the imaginary axis in a continuous piecewise linear system. This phenomenon is called focus-center-limit cycle bifurcation which resembles Andronov-Hopf bifurcation of differentiable systems. The Jacobian matrix must have a real eigenvalue and a pair of complex eigenvalues, which are pure imaginary and form a conjugate pair at the critical parameter values. Therefore, appearance of a linear center is expected when the focus-center-limit cycle bifurcation occurs. In the phase space, it can be seen that the equilibrium point is a focus before the bifurcation occurs. For the critical parameter values, it becomes a linear center which is limited by a separation plane, then a limit cycle bifurcates from the most external periodic orbit of the center passing through another linear region, and the stability of the focus changes. This transition can be clearly observed in the case examined in Section 3.3.3. Detailed description of the focus-center-limit cycle bifurcation in planar and 3-dimensional piecewise linear systems can be found in studies of Freire et al. (1997); Freire (2005), and book of Ponce et al. (2022) summarizes the results related to the FCLC bifurcation.

The focus-center-limit cycle bifurcation can be called supercritical when it results in birth of a stable limit cycle, and subcritical, if the periodic orbit is unstable, like in case of Andronov-Hopf bifurcation of differentiable dynamics (Perko 2013). Along the focus-center-limit cycle bifurcation, a higher codimension bifurcation can appear which is called degenerate focus-center-limit cycle bifurcation in book of Ponce et al. (2022). This can be observed when the bifurcation changes from supercritical to subcritical, similarly as the Bautin bifurcation appears in differentiable systems. It is known, that in smooth systems there is a bifurcation curve originating at the Bautin point, called fold bifurcation of cycles, at which a stable and an unstable limit cycle collide and disappear (Kuznetsov 1998). In piecewise linear systems, fold bifurcation of cycles can also appear when degenerate focus-center-limit cycle bifurcation occurs, as it is described by Ponce et al. (2022).

#### Bifurcation diagrams of periodic orbits

The necessary conditions for the focus-center-limit cycle bifurcation were given generally in Section 3.1 in formula (4) for the steady state of Case 3, and in (5) and (6) for Case 4. This means that the *β* value at which the bifurcation occurs can be determined for any chosen values of the other 10 parameters. At this *β* value, the stability of the affected equilibrium point changes and a periodic orbit is born or disappears which is stable in case of a supercritical bifurcation and unstable if the bifurcation is subcritical. Now the bifurcation diagrams are studied in the parameter plane $\left( c,\beta \right)$ while the value of connection parameter $J_{EE}$ is also varied. The remaining parameters are fixed at

15 $$\begin{aligned}&g_E=1, g_I=4, \theta _E=-5, \theta _I=25, \nonumber \\&J_{EI}=1.5, J_{IE}=10, J_{II}=0.5, \tau _E=7, \tau _I=2, \tau _a=90. \end{aligned}$$

These values are partly chosen from the study of Jercog et al. (2017), but here we have introduced a few changes that result in richer dynamical behaviour. For instance, changing the value of $J_{EI}$ from 1, which was applied by Jercog et al. (2017), to 1.5, enables the FCLC bifurcation to occur in the case of the equilibrium point corresponding to Case 3. Modifying two of the three time constants, which were originally $\tau _E=10,\tau _a=500$ in the work of Jercog et al. (2017), plays an important role in the occurrence of ripple-like oscillations, which will be presented in a later section. In particular, the adaptation time constant is reduced to $\tau _a=90$, which remains within the biologically plausible range of spike-frequency adaptation timescales Benda and Herz (2003). Here we have fixed the value of $\theta _E$ to *-5*, while the value of $J_{EE}$, *c* and *β* are varied.

**Fig. 4:**
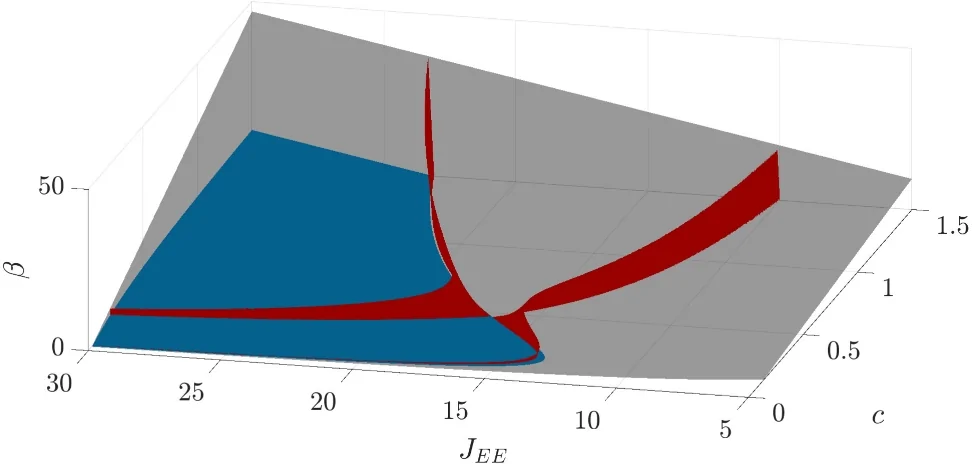
Surface of focus-center-limit cycle bifurcation for steady state of Case 4 (red) bounded by surface of (11) (blue) and surface of (10) (semi-transparent black). The bifurcation diagram was plotted for the fixed parameter values listed in equation (15)

The formula for the focus-center-limit cycle bifurcation occurring for steady state of Case 3 determines a vertical line in the $\left( c,\beta \right)$ plane with the equilibrium point stable on the left side and unstable on the right side. The bifurcation curve is more interesting for the steady state corresponding to Case 4 separating several domains according to stability. In order to list all possible 2-dimensional bifurcation diagrams that may occur, the 3-dimensional diagram of Case 4 is plotted according to formulas (5) and (6). Focus-center-limit cycle bifurcation forms the red surfaces in Fig. 4 which are bounded by the blue surface of (11) from below and by the semi-transparent black surface of (10) from above.

**Fig. 5:**
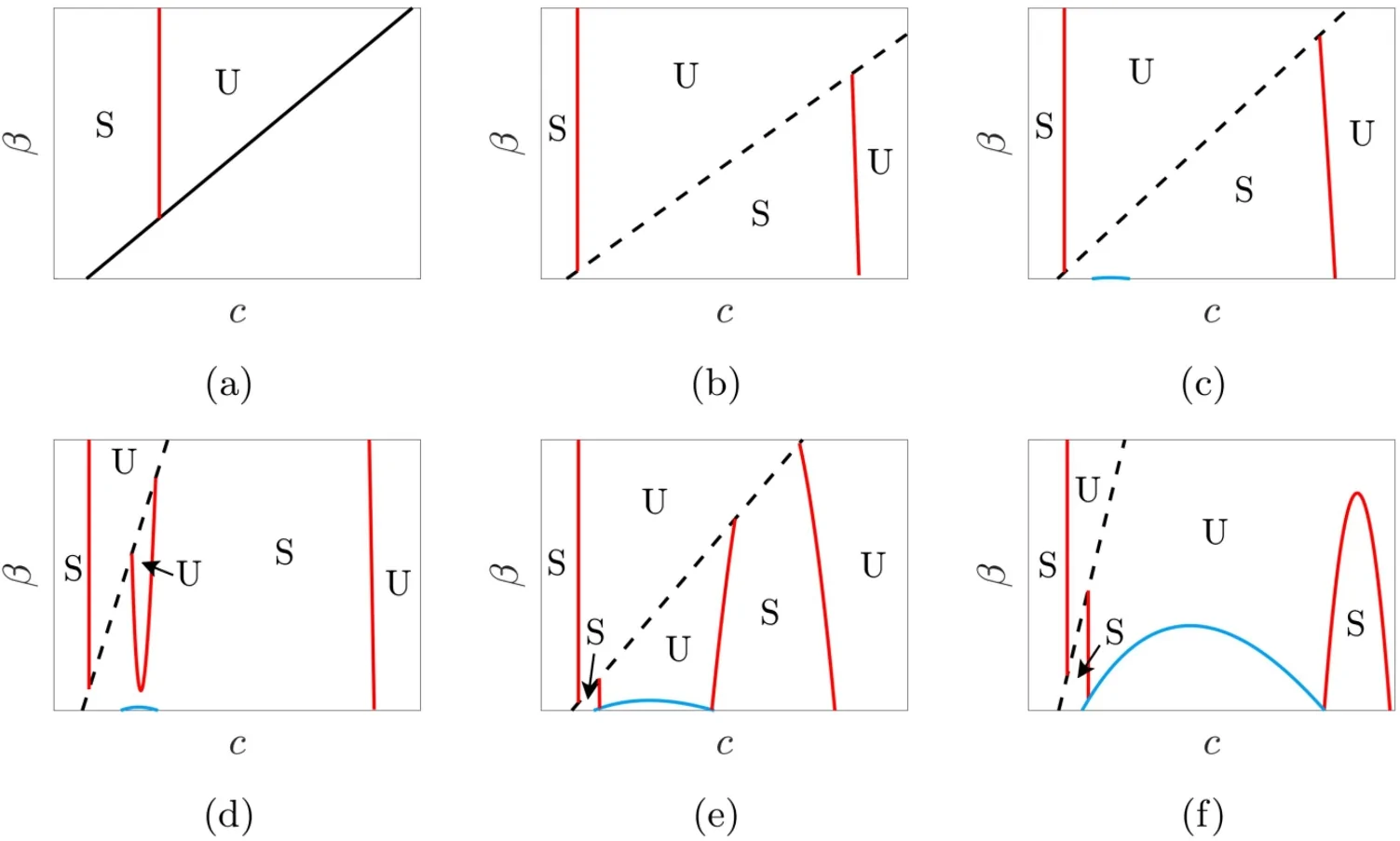
Bifurcation diagrams for fixed parameter values listed in equation (15) and (a) $J_{EE}=5$, (b) $J_{EE}=11.4$, (c) $J_{EE}=13.72$, (d) $J_{EE}=13.79$, (e) $J_{EE}=15$, (f) $J_{EE}=16$. Solid black and blue curves show the change in the number of equilibria given by equations (10) and (11), respectively. Dashed lines indicate transitions between Cases 1-4, which the steady states are connected to. Red curves show the focus-center-limit cycle bifurcation. Letters S and U denote whether the equilibrium point is stable or unstable in each domain

Now we find those values of $J_{EE}$ that lead to different bifurcation diagrams in the $\left( c,\beta \right)$ plane. Figure 5 shows that 6 different 2-dimensional bifurcation diagrams can be listed as the value of $J_{EE}$ is varied. The FCLC bifurcation curve of equilibrium corresponding to Case 3 is also plotted in the 2-dimensional diagrams. In these figures, focus-center-limit cycle bifurcation curves are plotted by red. Curve (11) is shown in blue, while curve (10) is plotted as a solid black line when it corresponds to a change in the number of steady states and as a dashed black line when it indicates a transition between Cases 1-4 without changing the number of steady states. With parameter values listed in equation (15), the system can have at most one equilibrium point in each domain where letters S and U denote that the steady state is stable or unstable. The birth of a periodic orbit via focus-center-limit cycle bifurcation is investigated in the following for a fixed value of $J_{EE}$.

**Fig. 6:**
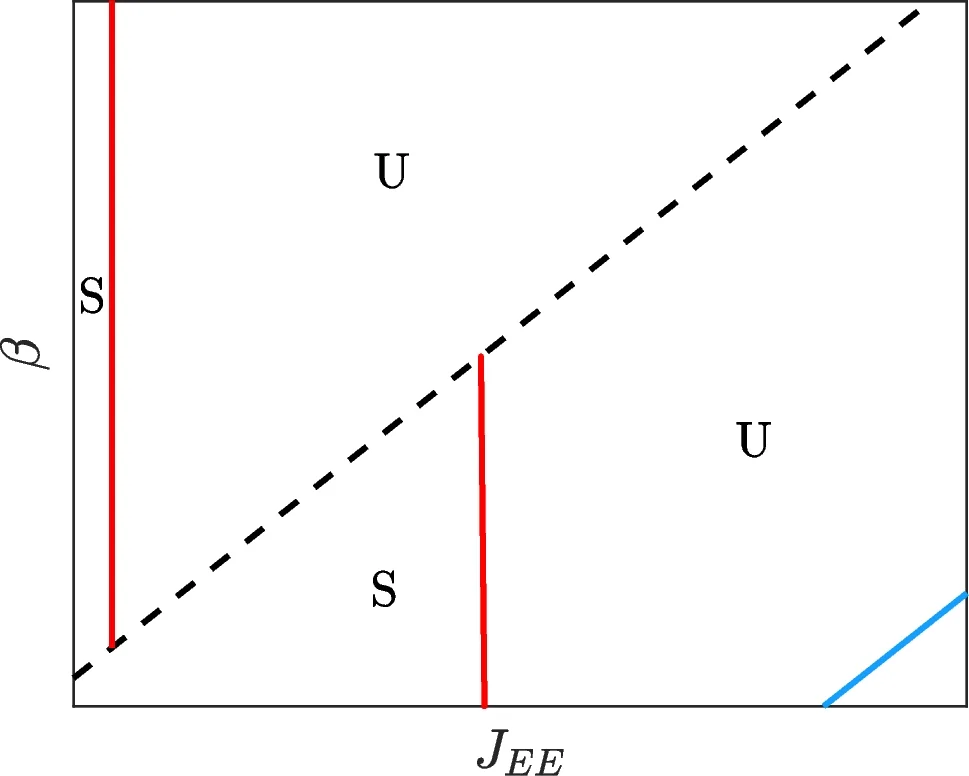
Bifurcation diagram for fixed parameter values listed in equation (15) and *c=1*. Blue curve shows the change in the number of equilibria given by equation (11). The dashed line indicates transitions between Cases 3 and 4, which the steady states are connected to. Red curves show the focus-center-limit cycle bifurcation. Letters S and U denote whether the equilibrium point is stable or unstable in each domain

For comparison, Figure 6 shows the bifurcation diagram obtained by varying $J_{EE}$ instead of *c*. This diagram corresponds to the intersection of the three-dimensional bifurcation diagram shown in Fig. 4 with the plane *c=1*. The resulting dynamics are qualitatively similar to those presented in Fig. 5b, which will be discussed in detail in the next section. The main difference is the presence of a parameter region where no equilibrium point exists.

#### FCLC bifurcation for a fixed parameter set

**Fig. 7:**
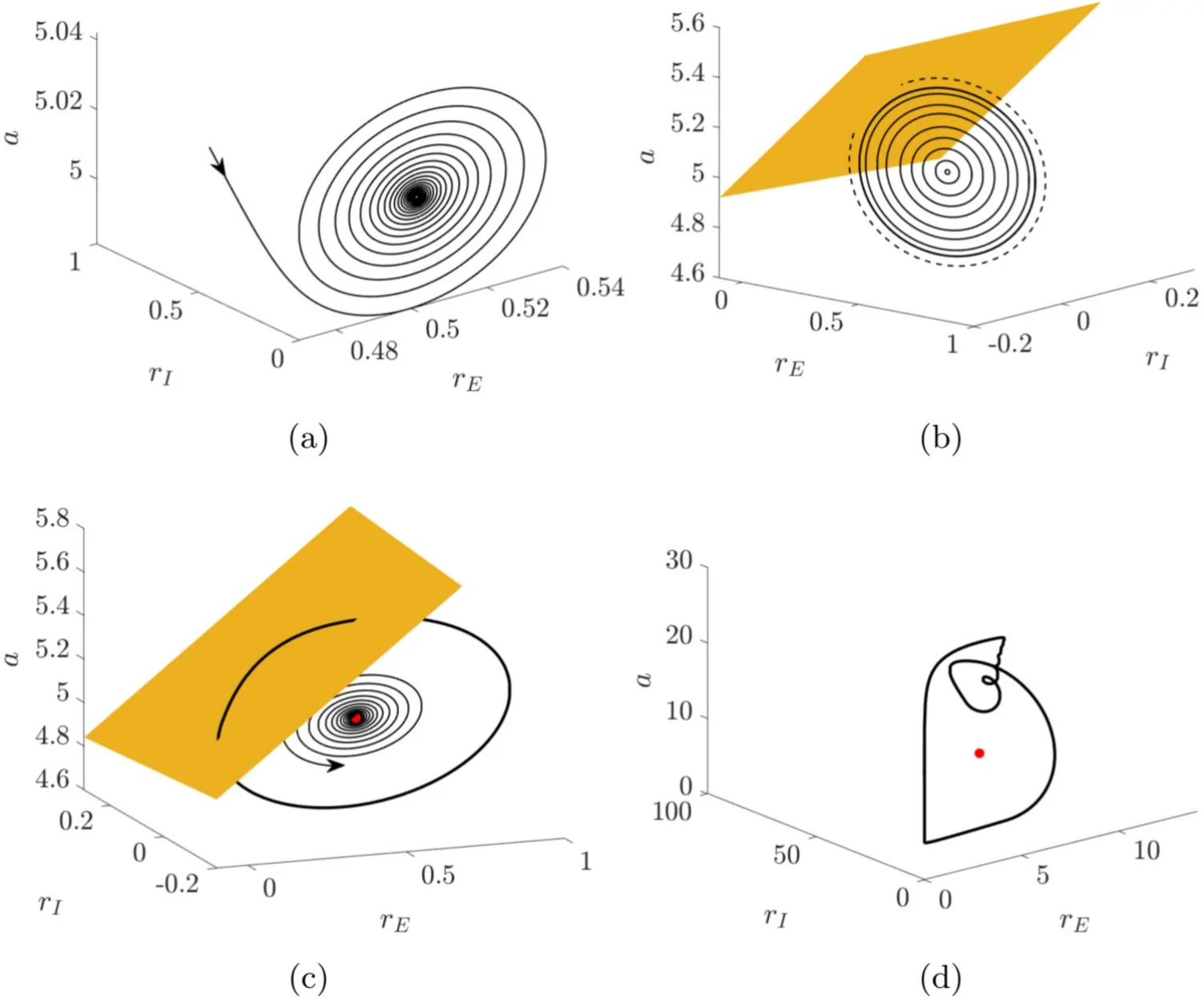
Birth of stable periodic orbit for $J_{EE}=11.4$ and *β =10* around the steady state of Case 3. The other fixed parameter values are listed in equation (15). The equilibrium is (a) a stable focus if *c=0.09*, (b) a linear center on the FCLC curve (*c=0.09454*), (c) unstable focus, and a stable limit cycle surrounds it which remains in the $(a,r_E)$ plane with $r_I=0$ in for *c=0.1*, (d) remains unstable, and the stable periodic orbit evolves in the 3-dimensional phase space

Now one of the six bifurcation diagrams shown in Fig. 5 is chosen to be studied in more detail by fixing the value of $J_{EE}$. If $J_{EE}$ is small, FCLC bifurcation can only occur in case of equilibrium corresponding to Case 3, while larger values of $J_{EE}$ lead to more complex bifurcation diagrams in case of steady state of Case 4. As a compromise between small and large values of $J_{EE}$, we show the bifurcation diagram corresponding to Fig. 5b and the same fixed parameter values are applied as listed in equation (15). In this case the system has 1 steady state above the dashed black line (10) which is the equilibrium point of Case 3. It is stable on the left side of the red line, denoting the focus-center-limit cycle bifurcation, but it loses its stability crossing it and a stable periodic orbit occurs around it. The process of the birth of this periodic orbit is the same as described in Section 3.3.1 and shown in Fig. 7. The equilibrium is a stable focus on the left side of the FCLC curve, while for those parameter values at which the bifurcation occurs, it becomes a linear center, which is limited by a separation plane. It can be observed, that close to the FCLC bifurcation curve, the periodic orbit remains in the equilibrium plane, namely the $\left( a,r_E\right)$ plane with $r_I=0$, reflecting the fact that the $r_I$ coordinate of the equilibrium point in Case 3 is also zero. Further away from the bifurcation curve, however, the periodic orbit departs from this plane and evolves in the full three-dimensional phase space, as shown in Fig. 7d."

**Fig. 8:**
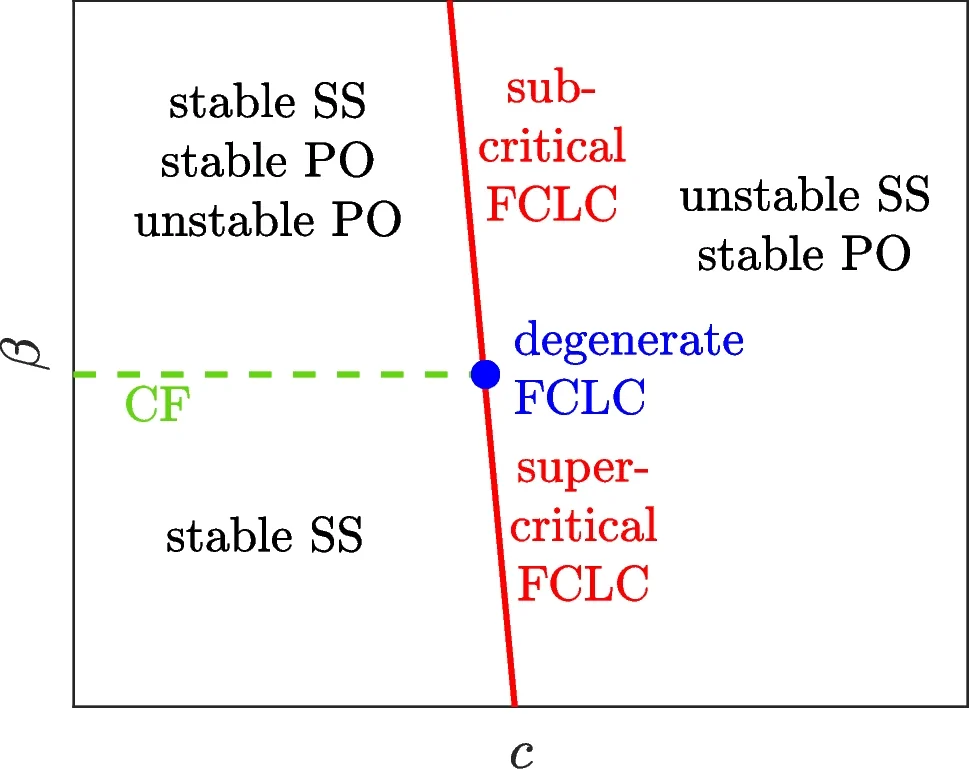
Bifurcation curves and phase portraits around the degenerate focus-center-limit cycle point for $J_{EE}=11.4$ and parameter values listed in equation (15). Steady states (SS), periodic orbits (PO), cycle fold bifurcation (CF) and focus-center-limit cycle bifurcation (FCLC) are denoted

**Fig. 9:**
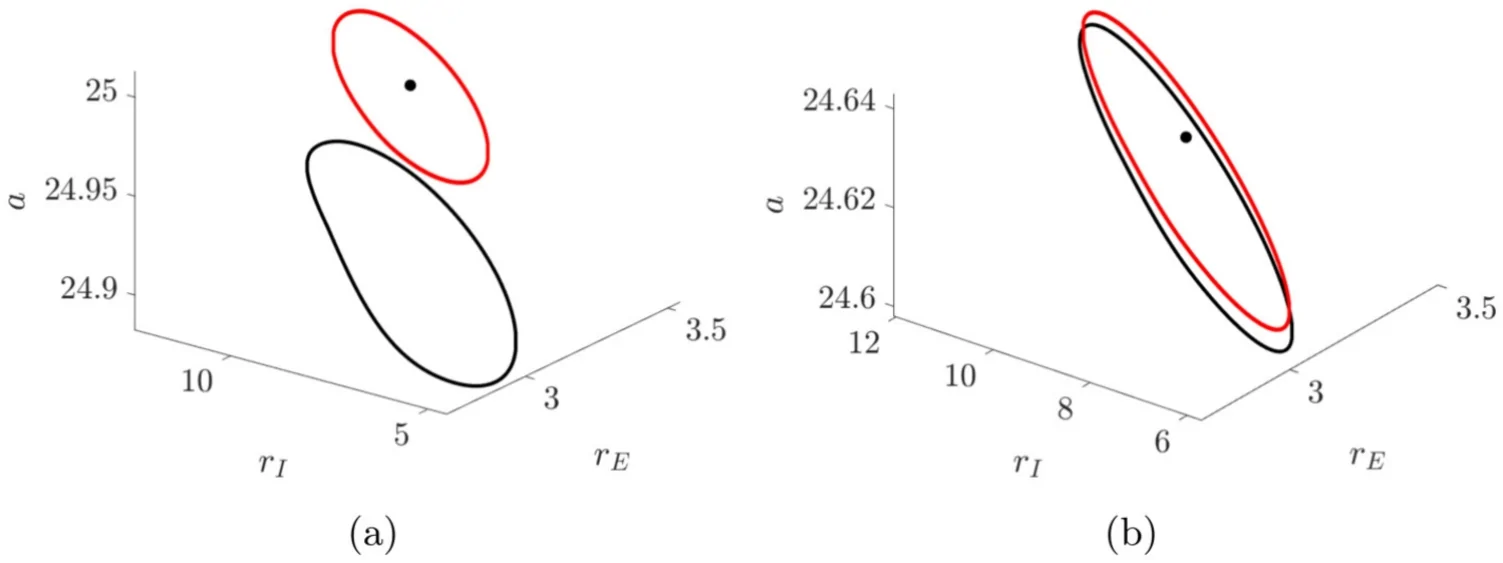
Stable (black) and unstable (red) periodic orbits are getting closer at (a) *β =8*, and (b) *β =7.785*, *c=1*, $J_{EE}=11.4$ and other fixed parameter values are listed in equation (15)

Below the dashed line in Fig. 5b, the system also has a single equilibrium point which corresponds to Case 4. This steady state is stable on the left side of the focus-center-limit cycle bifurcation curve and unstable on the right. Now the stability of the periodic orbit that appears due to focus-center-limit cycle bifurcation, depends on where it is born along the bifurcation curve. The bifurcation is supercritical in the bottom part of the curve, resulting in a birth of a stable periodic orbit around the unstable steady state on the right. By increasing the value of *β* the bifurcation becomes subcritical leading to the birth of an unstable periodic orbit around the stable steady state on the left side of the focus-center-limit cycle curve. This change indicates that there must be a degenerate focus-center-limit cycle bifurcation bifurcation point somewhere along the red curve, similarly to the Bautin bifurcation of smooth systems. Studying the phase portraits of system (1) it turns out that there is also a cycle fold bifurcation curve starting from the degenerate FCLC point. On the left side of the supercritical part of the focus-center-limit cycle bifurcation, system (1) has a single steady state which is globally stable, as it is shown in Fig. 8. Passing the bottom part of focus-center-limit cycle bifurcation curve, the equilibrium loses its stability and a stable periodic orbit occurs. Then passing the subcritical (upper) part of the focus-center-limit cycle bifurcation curve results in a birth of an unstable limit cycle, and the steady state becomes stable. In this domain of the parameter plane, the stable and the unstable periodic orbit coexist and getting closer if the value of *β* is decreased (see Fig. 9) until they collide and disappear as a result of saddle-node bifurcation of cycles. We note that the unstable periodic orbit was determined by constructing the Poincaré map on the section $\Sigma =\{(r_E,r_I,a):r_I=r_I^*\}$, where $r_I^*$ is the $r_I$-coordinate of the equilibrium point. Trajectories starting from points on the section were computed numerically and followed until their first return to the section, where each intersection point was also determined numerically, and then searching for a periodic point that is repelling in a certain direction. The coexisting periodic orbits with the stable steady state are shown in Fig. 9 for two different values of *β*. Bifurcation curves and phase portraits around the degenerate focus-center-limit cycle bifurcation point are sketched in Fig. 8.

**Fig. 10:**
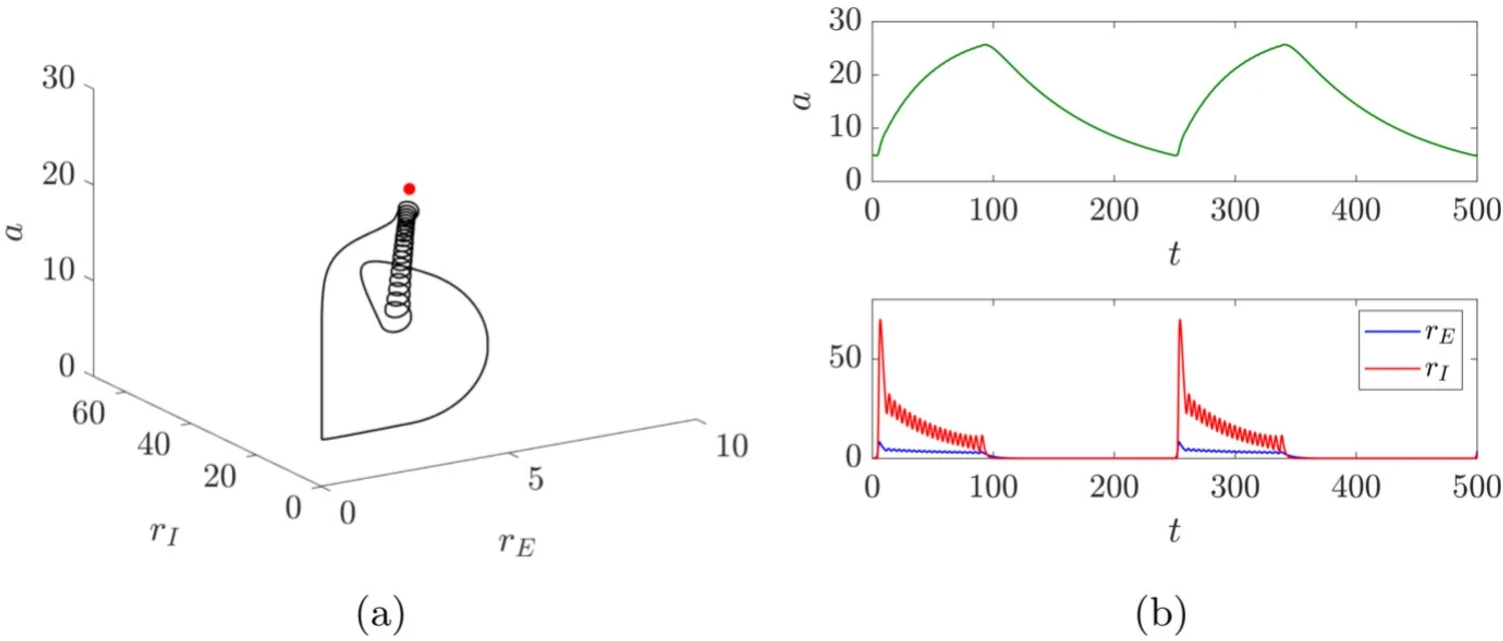
Sharp-wave ripple oscillation: (a) phase portrait of stable periodic orbit and unstable steady state, (b) adaptation (green) and firing rates ($r_E$: blue, $r_I$: red) in function of time, at *β =11*, *c=1.05*, $J_{EE}=11.4$ and other parameters fixed as listed in equation (15)

For larger values of *β*, on both sides of the focus-center-limit cycle curve, an interesting phenomenon can be detected that resembles a sharp-wave ripple (SWR) oscillation. This is a large periodic orbit with a slower part referred to as sharp waves, when only adaptation drives the system, and a fast-frequency oscillation within it during UP state, called ripple oscillation. In Fig. 10 sharp-wave ripple oscillation is shown in the 3-dimensional phase space and also the adaptation (green) and rates ($r_E$ with blue, $r_I$ with red) as a function of time. The oscillatory bursts observed in the model are qualitatively similar to hippocampal sharp-wave ripple events experimentally recorded from transverse ventral hippocampal slices (see, e.g., Fig. 10 in Buzsáki (2015)), where transient bursts of high-frequency activity occur during sharp-wave events. Although the model is not intended to reproduce the detailed biological mechanisms underlying SWRs, it demonstrates that adaptation and excitatory-inhibitory interactions can generate ripple-frequency activity.

## Discussion

In this work, we aimed to provide a complete characterization of the parameter space of a population-based rate model (1) in terms of its dynamical behaviour. The model, originally introduced by Jercog et al. (2017), describes the mean firing activity of an excitatory and an inhibitory population recurrently connected. Negative feedback is also added to the excitatory population, which represents the firing adaptation experienced by excitatory cells.

Due to the fact that the activation function is a threshold linear function, the model can be examined using analytical tools. In this paper, all equilibrium points and conditions for their existence were calculated, and comprehensive stability analysis was presented. Fold bifurcation curves were determined indicating changes in the number of steady states and all possible bifurcation diagrams were listed that may occur for a given parameter setting. We also investigated how focus-center-limit cycle bifurcation can occur in the system, resulting in stability change of an equilibrium and a birth of a periodic orbit. Analytical formulas were derived for the bifurcation curves, however bifurcation diagrams were listed varying only the value of three control parameters. The dynamical behaviour of the system was studied in a two-dimensional parameter space in detail, and a codimension-two bifurcation was also detected. The most interesting phase portraits were presented including the birth of a limit cycle via focus-center-limit cycle bifurcation and also periodic behaviour resembling sharp-wave ripple oscillation.

These results show that a wide variety of bifurcation diagrams can be distinguished by varying the full set of 11 parameters, focusing only on the number of equilibrium points. When periodic solutions are included and only a subset of parameters is treated as control parameters, the resulting dynamical behaviour becomes substantially more complex. Detection of further bifurcations leading to oscillatory dynamics could be an interesting direction of future work.

## Data Availability

No datasets were generated or analysed during the current study.

## Appendix A

### A.1 Detailed description of the 8 domains in the $(\theta _E,\theta _I)$ plane

Here the 8 domains of the $\left( \theta _E,\theta _I\right)$ plane in Fig. 1 are examined one by one, showing the proof of Theorem 9. We use that only $\theta _E$ and $\theta _I$ can take positive and negative values, while all the other parameters are positive. Bifurcation curves defined by equations (7), (10) and (11) will be referred to as (7), (10) and (11), respectively. They form the bifurcation diagrams (see Theorem 8), which have a common point $\left( \dfrac{\theta _E}{g_Ib_I};\dfrac{b_E\theta _E}{g_Ib_I\theta _I}-\dfrac{1}{g_E}\right)$. (10) and (11) have an other intersection too at $\left( 0;-\dfrac{1}{g_E}\right)$. We also use that the curve given in equation (11) is concave for all *c≥ 0*.

I. $\theta _E>0$, $\theta _I>0$, $b_E>0$, $b_I>0$ The steady state of Case 1 exists for all *c,β>0* according to Proposition 1, equilibrium of Case 3 exists below the line given in equation (10), whose slope is positive. As $\theta _I>0$ here, steady state of Case 2 can not exist, see Proposition 2. On the left side of (7) the steady state corresponding to Case 4 exists above (10) but below curve (11), while on the right side of (7) it exists below (10) but above (11). Investigating the position of the bifurcation curves results in 4 different bifurcation diagrams. Figure 2a shows the case when (11) take only negative values for all *c≥ 0*. Figure 2b and 2c presents that (11) takes positive values only on the right and left side of the common point of the bifurcation curves, respectively. In Fig. 2d is shown when steady state of Case 4 exists on both side of (7).
II. $\theta _E>0$, $\theta _I>0$, $b_Eb_I<0$ Here we have 2 cases according to the sign of $\det \tilde{J}$.
  (i) $\det \tilde{J}>0$, as then $b_E>0$, $b_I<0$ The steady state of Case 1 exists for all *c,β>0* as $\theta _E, \theta _I>0$, equilibrium of Case 3 exists below the line given in equation (10), whose slope is positive. Equilibrium of Case 2 does not exist because $\theta _I$ is positive. Now (7) is located at *c<0*. The steady state corresponding to Case 4 exists above line (10) but below (11). These conditions lead to a single possible bifurcation diagram shown in Fig. 2e.
  (ii) $\det \tilde{J}<0$, then $b_E<0$, $b_I>0$ The steady state of Case 1 exists for all *c,β>0* and steady state of Case 2 does not exist, see Propositions 1-2. Now, equilibrium corresponding to Case 3 can not exist, as conditions (8) can not be satisfied because of the negativity of $b_E$. Steady state of Case 4 exists above (10) and curve (11) on the left side of (7). The slope of line given in equation (10) is negative and it takes $-\dfrac{1}{g_E}$ at *c=0*, therefore this line is located in the fourth quadrant for *c≥ 0*. These conditions result in two different bifurcation diagrams. Figure 2f shows when (11) can not be positive, thus the only equilibrium of the system is of Case 1. In Fig. 2g the steady state of Case 4 also exists below (11). (7) can not border any domain as at the common point of the three bifurcation curves *β <0*.
III. $\theta _E>0$, $\theta _I>0$, $b_E<0$, $b_I<0$ This case is quite similar to the previous one. The only difference is that (7) is located at *c<0* and equilibrium of Case 4 exists on the right side of it. The bifurcation diagrams we get are also the same, namely Fig. 2f and 2g.
IV. $\theta _E>0$, $\theta _I<0$ The sign of $\theta _E>0$ and $\theta _I<0$ determine that $b_E,b_I<0$. Steady state of Case 1 and 3 can not exist as $\theta _I<0$. Equilibrium of Case 2 exists on the right side of (7) (see Proposition 2) which is located at *c<0*, therefore equilibrium of Case 2 exists for all *c,β>0*. Steady state of Case 4 exists below (10) and (11) resulting in two different bifurcation diagrams. Figure 2f presents when curve given in equation (11) takes only negative values for all *c>0*, therefore the only equilibrium is of Case 2. When (11) takes positive values too, the bifurcation diagram is shown in Fig. 2g. It can be noticed that (10) and (11) do not have any common point for *c>0*, so their slope at *c=0* determine that (11) runs above (10) for all *c>0*.
V. $\theta _E<0$, $\theta _I<0$, $b_E<0$, $b_I<0$ Steady states corresponding to Cases 1 and 3 can not exist here, see Proposition 1 and conditions (8)-(9). Equilibrium of Case 2 exists on the right side of (7), where steady state of Case 4 exists below (10) and (11), but on the left it exists above (10) and (11). These conditions lead to four different bifurcation diagrams. Figure 2j shows when (11) takes only negative values for all *c>0*. Figures 2k and 2h presents that for some parameter values, (11) takes positive values only on the left or right side of (7), respectively. Figure 2i shows the most complicated bifurcation diagram with these conditions where domains with different number of steady states are formed by (11) and line (7) together.
VI. $\theta _E<0$, $\theta _I<0$, $b_Eb_I<0$ Here we have 2 cases according to the sign of $\det \tilde{J}$.
  (i) $\det \tilde{J}>0$, then $b_E>0$, $b_I<0$ Steady states corresponding to Cases 1 and 3 can not exist here, see Proposition 1 and conditions (8)-(9). Equilibrium of Case 2 exists on the right side of (7). On the left side of it steady state of Case 4 exists above (11). Line given in equation (10) is not located in the first quadrant of the parameter plane as its slope is negative. As a result of these conditions we get two bifurcation diagrams that differ in whether (11) takes on any positive values or not, shown in Figs. 2k and 2j, respectively.
  (ii) $\det \tilde{J}<0$, then $b_E<0$, $b_I>0$ Steady states of Cases 1-3 can not exist under these conditions according to Propositions 1-3. Equilibrium of Case 4 exists above (10) and (11), where (11) is located above (10) for all *c>0*. Now there is only one possible bifurcation diagram which is shown in Fig. 2l.
VII. $\theta _E<0$, $\theta _I<0$, $b_E>0$, $b_I>0$ According to Proposition 1, steady state corresponding to Case 1 can not exist here, just like the steady state of Case 3, as none of the conditions (8)-(9) can be satisfied. Line (7) is located at *c<0*, therefore equilibrium of Case 2 does not exist as it would exist on the left side of line (7). The slope of line (10) is negative, then it does not intersect the first quadrant of the parameter plane. Steady state of Case 4 is the only one that can exist, and the condition for it is to be located above curve (11). Two bifurcation diagrams can be detected here, when (11) takes only negative values which can be seen in Fig. 2f, and when (11) takes positive values too and below it there is no steady state, shown in Fig. 2l.
VIII. $\theta _E<0$, $\theta _I>0$ The sign of $\theta _E<0$ and $\theta _I>0$ determine that $b_E,b_I>0$. Steady state of Case 1 and 2 can not exist, see Propositions 1-2. The slope of line given in equation (10) is positive and the steady state of Case 3 exists above this line according to condition (9). Line defined in equation (7) is located at *c<0*, and equilibrium corresponding to Case 4 exists on the right side of it, below line (10) but above (11). These conditions result in two different bifurcation diagrams. Figure 2m shows when (11) does not take any positive values for all *c>0*, therefore the system has one steady state for any *c,β* values chosen from the parameter plane, which corresponds to Case 1 above (10) and to Case 3 below it. In Fig. 2n it can be seen that (11) takes positive values too, so another domain appears formed by (11), where the system has no equilibrium point.
